# Nr0b1 is a negative regulator of *Zscan4c* in mouse embryonic stem cells

**DOI:** 10.1038/srep09146

**Published:** 2015-03-16

**Authors:** Setsuko Fujii, Satomi Nishikawa-Torikai, Yoko Futatsugi, Yayoi Toyooka, Mariko Yamane, Satoshi Ohtsuka, Hitoshi Niwa

**Affiliations:** 1 Laboratory for Pluripotent Stem Cell Studies, RIKEN Center for Developmental Biology, 2-2-3 Minatojima-minamimachi, Chuo-ku, Kobe 650-0047, Japan; 2 Division of Embryology, National Institute for Basic Biology (NIBB), Okazaki 444-8787, Japan; 3 Laboratory for Development and Regenerative Medicine, Kobe University Graduate School of Medicine, 7-5-1 Kusunokicho, Chuo-ku, Kobe 650-0017, Japan; 4 JST, CREST, Sanbancho, Chiyoda-ku, Tokyo 102-0075, Japan

## Abstract

*Nuclear receptor subfamily 0, group B, member 1* (*Nr0b1*, also known as *Dax1*) is regarded as an important component of the transcription factor network that governs pluripotency in mouse embryonic stem (ES) cells. Here we generated inducible knockout ES cells for *Nr0b1* using the Cre-*loxP* system and analyzed its precise function. We succeeded in establishing the *Nr0b1*-null ES cells and confirmed their pluripotency by showing their contribution to chimeric embryos. However, they proliferated slowly with over-expression of 2-cell stage specific transcripts including *Zscan4c*, which is known to be involved in telomere elongation in ES cells. We revealed that over-expression of *Zscan4c* prevents normal self-renewal by inducing arrest at G2 phase followed by cell death and that Nr0b1 directly represses the *Zscan4c* promoter. These data indicated that *Nr0b1* is not essential to maintain pluripotency but is involved in the proper activation of 2-cell specific transcripts for self-renewal.

*Nr0b1* (also known as *Dosage-sensitive sex reversal-adrenal hypoplasia congenital on the X-chromosome gene-1: Dax1*) is a unique member of the nuclear family because it lacks the DNA binding domain and works to modulate the function of other nuclear receptors^1^. It has been well analyzed that *Nr0b1* is involved in germ cell development^2^,^3^. *Nr0b1* is expressed at a high level in ES cells but not in epiblast stem cells (EpiSCs), suggesting the role in the naïve state of pluripotency^4^. The expression of *Nr0b1* is regulated by the LIF signal via Jak-Stat3 pathway^5^ as well as by Oct3/4^5^, Nanog^6^, Nr5a2^6^ and Esrrb^7^. Conversely, Nr0b1 binds to Oct3/4 to inhibit its transcriptional activity and the over-expression of *Nr0b1* induces differentiation toward trophectoderm as in the case of *Oct3/4* knockout^8^. It was also reported that Nr0b1 interacts with Esrrb to inhibit its transcriptional activity^7^. Interestingly, while the biochemical analyses suggested that Nr0b1 interacts with Nr5a2 to suppress its function^9^,^10^, it was also demonstrated that Nr0b1 cooperates with Nr5a2 and Steroid receptor RNA activator 1 (Sra1) to activate the *Oct3/4* promoter^11^. What happens if *Nr0b1* function is eliminated in ES cells? *Niakan et al* reported that either knock-down of *Nr0b1* by siRNA or knock-out of *Nr0b1* by the Cre-*lox* system induces differentiation of ES cells^12^. This is consistent with the second report by *Khalfallah et al* showing that siRNA-mediated knock-down of *Nr0b1* causes multi-lineage differentiation^13^. However, in both cases, the primary effect of the withdrawal of *Nr0b1* on the pluripotency-associated transcription factor network was not well analyzed. Here we generated an inducible knockout ES cell line of *Nr0b1* with the Cre-*lox* system to examine its precise role to regulate the transcription factor network. Our data indicated that *Nr0b1* is dispensable for maintaining pluripotency but is involved in the transcriptional regulation of 2-cell specific genes in ES cells.

## Results

### Establishment of inducible knockout ES cell lines for *Nr0b1*

**Figure 1:**
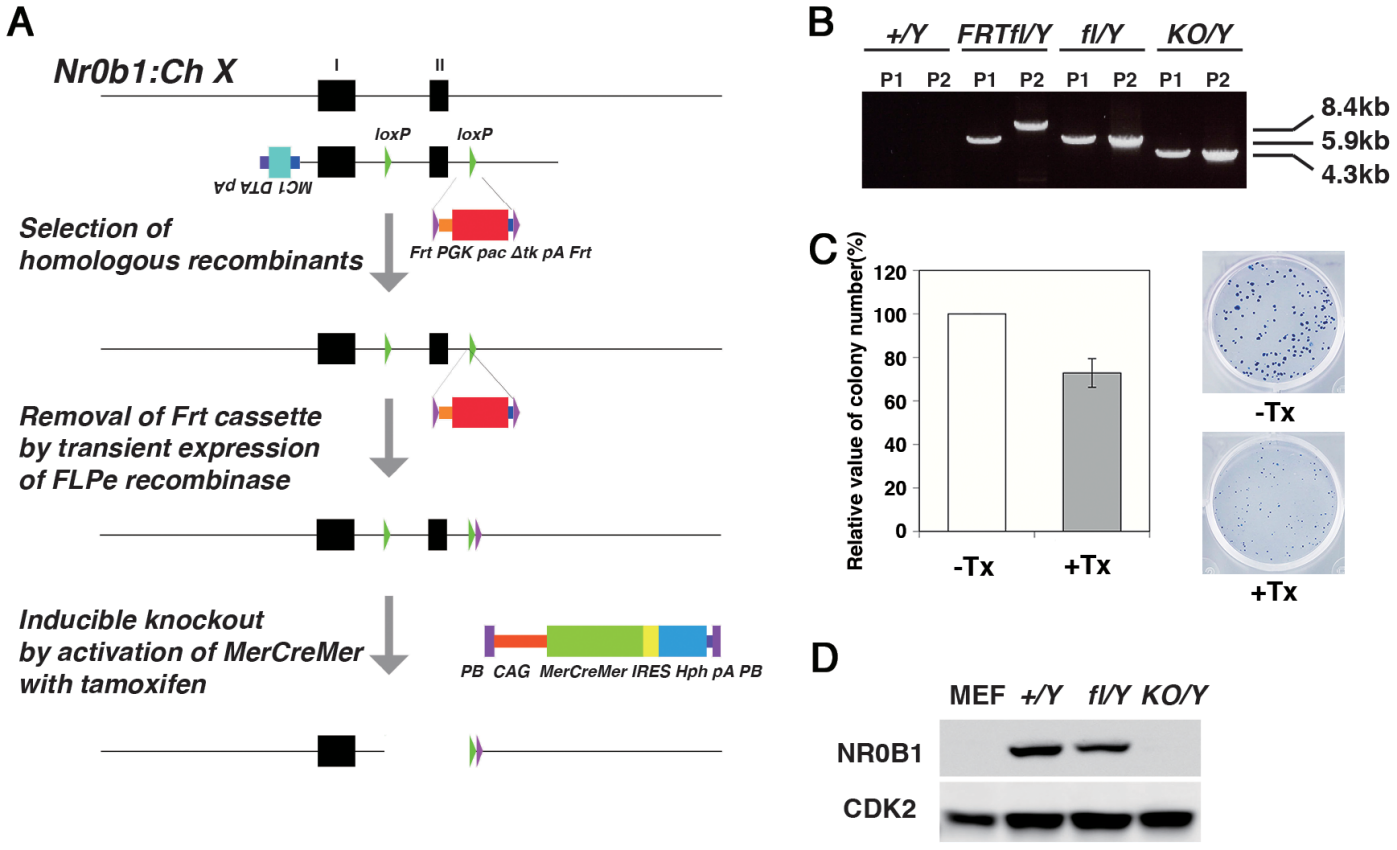
Generation of inducible *Nr0b1*-null ES cells. (a) Schematic representation of the strategy to generate the inducible *Nr0b1*–null ES cells. (b) PCR genotyping of the ES cells at each step of genetic engineering. P1 and P2 were the results of PCR with primer pairs KO PCR 1 and 2, respectively. (c) Colony formation of the inducible *Nr0b1*-null ES cells. The stem cell colonies were scored by the compact morphology after Leischman staining. Error bars indicate standard deviation (n = 3). (d) Depletion of NR0B1 protein in the *Nr0b1*-null ES cells determined by western blotting.

The *Nr0b1* gene consists of 2 exons (Fig. 1a). It has been reported that the deletion of exon 2 results in functional ablation. We made a knockout vector in which two *loxP* sites were inserted in intron 1 and the 3′ of exon 2, with a *PGKpac*Δ*tkpA* cassette flanked by *Frt* sites inserted adjacent to the 5′ end of the 3′ *loxP* site (Fig. 1a). The linearized knockout vector was introduced into male ES cells by electroporation followed by the selection with puromycin. As a result we obtained multiple clones with correct homologous recombination event confirmed by combinations of long-range genomic PCR (Fig. 1b), designated as *Nr0b1^flFrt/Y^* ES cells. Then the expression vector of the FLP recombinase (FLPe)^14^ was transiently transfected by lipofection followed by the selection with gancyclovir, resulting in the generation of ES cells in which the *PGKpac*Δ*tkpA* cassette was excised, designated as *Nr0b1^fl-/Y^* ES cells. Then the piggy-bac vectors for constitutive expression of hormone-inducible Cre (*MerCreMer*)^15^ and *Egfp* were introduced into the *Nr0b1^fl-/Y^* ES cells to obtain inducible *Nr0b1* knockout (*Nr0b1^KO/Y^*) ES cells.

The *Nr0b1^fl-/Y^* ES cells carrying *MerCreMer* and *Egfp* continued self-renewal as wild-type ES cells and contributed to chimeric embryos after blastocyst injection (see below). When these ES cells were treated with 4-hydroxy tamoxifen (Tx) to activate the Cre recombinase for 2 days followed by dissociation into single cells and plating onto a fresh culture, the colonies with typical ES cell morphology were formed after 6 days at a comparable rate (72%) to that of *Nr0b1^fl-/Y^* ES cells without the Tx treatment (Fig. 1c). Then these colonies were isolated and the genotype was examined by PCR. Among the 44 clones examined, 30 clones (68%) were genotyped as *Nr0b1^KO/Y^* ES cells that possess the deleted allele by the excision of the *floxed* region (data not shown), indicating that the loss of *Nr0b1* has no primary impact on the clonogenicity of ES cells. Finally we succeeded to establish multiple *Nr0b1^KO/Y^* ES cell lines in which proper loss of Nr0b1 protein was confirmed by western blot analysis (Fig. 1d and Supplemental Fig. 1). These data clearly indicated that the function of *Nr0b1* is dispensable for continuous self-renewal of ES cells in contrast to previous reports^12^,^13^.

### *Nr0b1* is required for proliferation of ES cells

**Figure 2:**
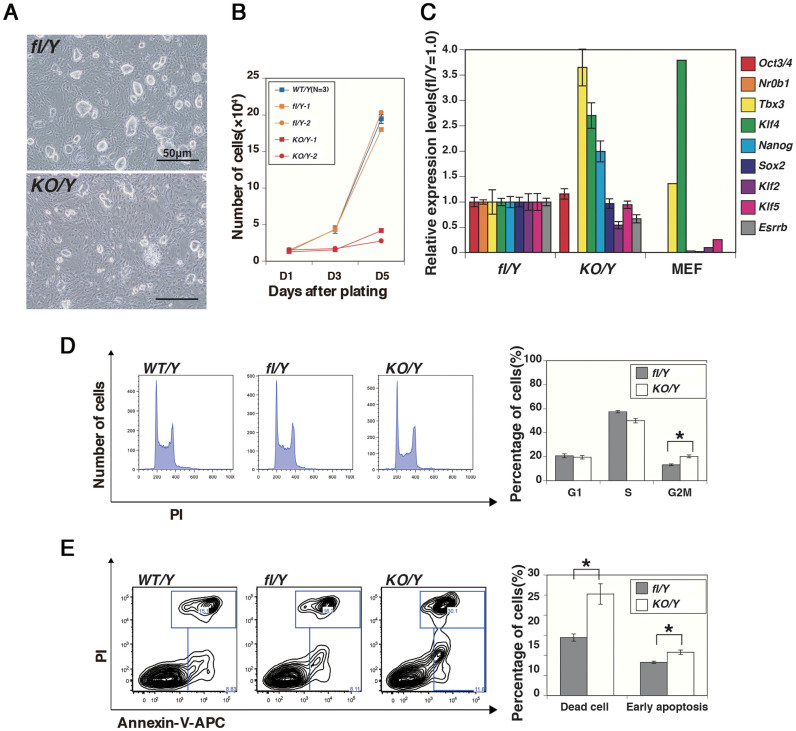
Defective proliferation of *Nr0b1*-null ES cells. (a) Colony morphologies of *Nr0b1^fl/Y^* and *Nr0b1^KO/Y^* ES cells after the culture for 5 days on feeder cells. (b) Proliferation ratio of wild-type (WT), *Nr0b1^fl/Y^* (fl/Y) and *Nr0b1^KO/Y^* (KO/Y) ES cells. 1 × 10^4^ cells were seeded on feeder cells and the numbers of cells were counted after 1, 3 and 5 days. Error bars indicate standard deviation (n = 3). (c) Quantitative RT-PCR analysis of *Nr0b1^fl/Y^* (fl/Y) and *Nr0b1^KO/Y^* (KO/Y) ES cells for the expressions of pluripotency-associated genes. The level of expression of each transcript in *Nr0b1^fl/Y^* ES cells was set at 1.0. Error bars indicate standard deviation (n = 3). (d) Cell-cycle profiling of wild-type (WT), *Nr0b1^fl/Y^* (fl/Y) and *Nr0b1^KO/Y^* (KO/Y) ES cells by FACS with propidium iodide (PI) staining. Proportions of the cells at each cell cycle were shown in the graph with error bars indicating standard deviation (n = 3). Asterisk indicates statistic difference (P < 0.05; t-test). (e) Analysis of early apoptosis and dead cells in wild-type (WT), *Nr0b1^fl/Y^* (fl/Y) and *Nr0b1^KO/Y^* (KO/Y) ES cells by FACS with Annexin-V and PI staining. Proportions of the dead cells and the cells at early apoptosis were shown in the graph with error bars indicating standard deviation (n = 3). Asterisk indicates statistic difference (P < 0.05; t-test).

During the establishment of *Nr0b1^KO/Y^* ES cells, we found that these ES cells formed smaller colonies than *Nr0b1^fl/Y^* and the wild-type (*Nr0b1^+/Y^*) ES cells at the same culture period (Fig. 2a). The measurement of the proliferation ratios of *Nr0b1^KO/Y^* ES cells revealed that it was less than 20% of *Nr0b1^fl/Y^* and *Nr0b1^+/Y^* ES cells (Fig. 2b). Although ES cells show decreased proliferation ratio when they undergo differentiation in vitro, *Nr0b1^KO/Y^* ES cells retained the expression of pluripotency-associated transcription factors such as *Oct3/4*^16^, *Sox2*^17^, *Nanog*^18^,^19^, *Klf4* and *Tbx3*^20^ (Fig. 2c) and kept the typical ES cell morphology (Fig. 2a). These data suggested that these *Nr0b1^KO/Y^* ES cells should retain proper pluripotency.

To reveal the reason for the slow proliferation of *Nr0b1^KO/Y^* ES cells, we examined the cell cycle profile by FACS analysis with propidium iodide (PI) staining of the DNA. ES cells have a characteristic cell-cycle profile, in which the cells at the G1 phase are few and those at the S phase are abundant in comparison to somatic cell types, reflecting the unlimited G1-S transition^21^. It has been reported that the proportion of G1 is increased in *Klf5*-null ES cells^22^ and *Sall4*-null ES cells^23^ that show slow proliferation. However, the proportion of the cells at the G1 phase was not altered in *Nr0b1^KO/Y^* ES cells (Fig. 2d). Instead, we found significant increase in the G2/M phase in *Nr0b1^KO/Y^* ES cells (Fig. 2d), suggesting that *Nr0b1* might modulate proliferation without affecting the G1-S transition.

Increased incidence of cell death could cause low proliferation ratio of ES cell population. Thus we tested the proportion of dead cells, and further examined whether the death was caused by apoptosis or not, by double-staining with PI and Annexin-V^24^. Early apoptotic cells are stained only by Annexin-V, whereas dead cells are stained by both. The proportions of the early apoptotic cells and also the overall dead cells were both increased in *Nr0b1^KO/Y^* ES cells (Fig. 2e). These data indicated that the function of *Nr0b1* might be involved in suppression of cell death caused by apoptosis.

### *Nr0b1*-null ES cells retain pluripotency

**Figure 3:**
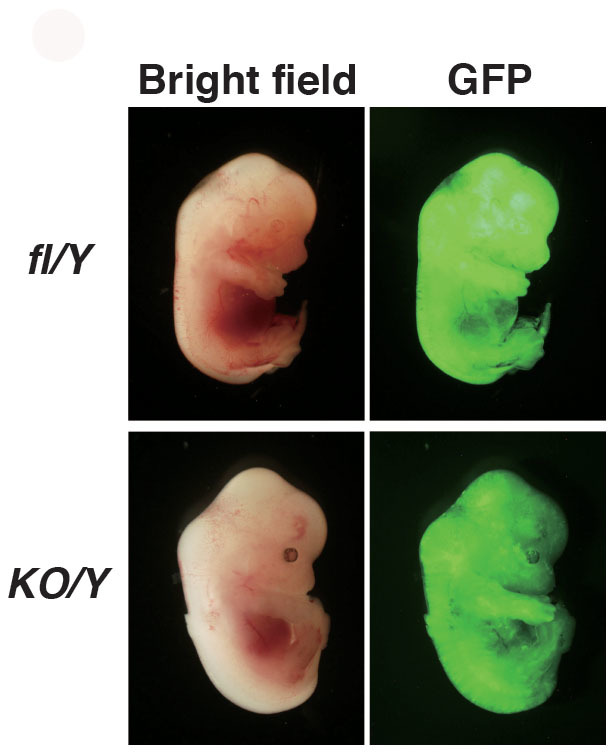
Chimeric embryos with *Nr0b1*-null ES cells. Chimeric embryos at 13.5 dpc obtained by injection of *Nr0b1^fl/Y^* (fl/Y) and *Nr0b1^KO/Y^* (KO/Y) ES cells carrying constitutively-active *Egfp* transgene.

Next we assessed whether *Nr0b1^KO/Y^* ES cells retain pluripotency. The dissociated *Nr0b1^KO/Y^* ES cells expressing *Egfp* were injected into blastocysts and their contribution in embryos dissected at E13.5 was evaluated. As shown in Figure 3, both *Nr0b1^fl-/Y^* and *Nr0b1^KO/Y^* ES cells contributed to chimera embryos as efficiently as *Nr0b1^+/Y^* ES cells. The efficiencies to give rise to chimeric emrbyos were also indistingishable between *Nr0b1^fl-/Y^* and *Nr0b1^KO/Y^* ES cells (Supplemental Table 2). Therefore, pluripotency is properly maintained in the absence of *Nr0b1* in ES cells.

### Aberrant expression of 2-cell specific genes in *Nr0b1*-null ES cells

**Figure 4:**
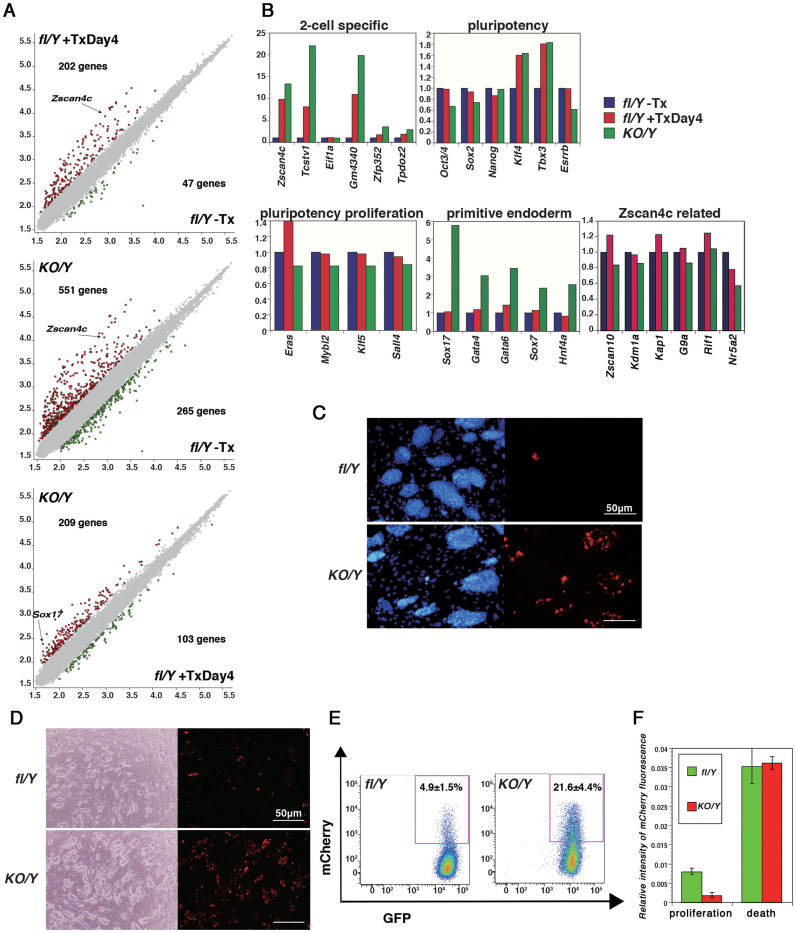
Up-regulation of 2-cell specific transcripts in *Nr0b1*-null ES cells. (a) DNA microarray analyses of *Nr0b1^fl/Y^* (fl/Y) ES cells cultured with or without tamoxifen (Tx) for 4 days, and *Nr0b1^KO/Y^* (KO/Y) ES cells. Scatter plots of log-ratios of relative expression levels were shown for each indicated pairs. The genes shown statistic difference (>2-fold, FDR = 0.05) were highlighted with red or green colors. (b) Highlight of the relative gene expressions of each category from the microarray data sets. (c) Immunostaining of *Nr0b1^fl/Y^* (fl/Y) and *Nr0b1^KO/Y^* (KO/Y) ES cells for Tcstv1. (d) Fluorescent photomicrograph of *Nr0b1^fl/Y^* (fl/Y) and *Nr0b1^KO/Y^* (KO/Y) ES cells carrying *pPB-Zscan4c-mCherry*. (e) FACS analysis of *Nr0b1^fl/Y^* (fl/Y) and *Nr0b1^KO/Y^* (KO/Y) ES cells carrying *pPBCAG-Egfp-IZ* and *pPB-Zscan4c-mCherry*. (f) Fluorescent intencity of mCherry in *Nr0b1^fl/Y^* (fl/Y) and *Nr0b1^KO/Y^* (KO/Y) ES cells carrying *pPB-Zscan4c-mCherry*. The cells undergoing proliferation and cell death were identified in live imaging and the fluorescent intensities were measured. The average intensities in each genotype are indicated with standard error.

The data shown above indicated that *Nr0b1* was required for accelerating cell proliferation and keeping normal mortality but dispensable for maintaining pluripotency. For further investigation of the role of *Nr0b1* in ES cells, we performed microarray analyses to examine the effect of *Nr0b1* elimination on the global gene expression. We compared the gene expression patterns of *Nr0b1^fl-/Y^* ES cells cultured with or without Tx for 4 days (*Nr0b1^fl-/Y^*+Tx and *Nr0b1^fl-/Y^*−Tx, respectively) and *Nr0b1^KO/Y^* ES cells, using the samples of the biological triplicates. The comparison between *Nr0b1^fl-/Y^*−Tx and *Nr0b1^fl-/Y^*+Tx was expected to show the primary effect of *Nr0b1* elimination, whereas that between *Nr0b1^fl-/Y^*+Tx and *Nr0b1^KO/Y^* would show the effect of the adaptive response to compensate the loss of *Nr0b1*. There was little difference in the gene expression patterns of wild-type and *Nr0b1^fl-/Y^*−Tx ES cells, indicating that the manipulation of *Nr0b1* as well as the expression of *MerCreMer* and *Egfp* had the minimal impact (data not shown). The comparison between the gene expression patterns of *Nr0b1^fl-/Y^*−Tx and *Nr0b1^fl-/Y^*+Tx ES cells showed characteristic differences. Among 202 genes significantly up-regulated in *Nr0b1^fl-/Y^*+Tx ES cells (Fig. 4a: >2-fold, FDR = 0.05), we found multiple genes categorized as 2-cell specific transcripts including *Zscan4c*, *Tcstv1*, and *Thoc4/Gm4340*^25^ (Fig. 4b). Among the pluripotency-associated genes, *Klf4* and *Tbx3* were slightly up-regulated, but the genes that have been previously reported to be associated with rapid proliferation (*Eras*^26^, *Mybl2*^27^,^28^, *Klf5*^22^ and *Sall4*^23^) did not alter their expression levels (Fig. 4b). In the comparison between *Nr0b1^fl-/Y^*+Tx and *Nr0b1^KO/Y^* ES cells, 209 genes were up-regulated and 103 genes were down-regulated (Fig. 4a). Interestingly, the differentiation markers for the endoderm (*Gata4, Gata6 Sox7, Sox17* and *Hnf4a*^29^) were over-expressed in *Nr0b1^KO/Y^* ES cells (Fig. 4b). The 2-cell specific transcripts remained over-expressed in *Nr0b1^KO/Y^* ES cells.

To confirm the over-expression of the genes identified by the microarray analysis, we performed immuno-staining for Sox7, Sox17, Gata4 and Tcstv1. Sox7, Sox17 and Gata4 were detected as a cluster in the colonies with differentiated cell morphology, suggesting that the increased expression level of those genes reflects the elevated incidence of spontaneous differentiation of *Nr0b1^KO/Y^* ES cells (Supplementary Fig. 2). *Tcstv1* (*2-cell-stage, variable group, member 1*) is known as one of the 2-cell specific transcripts expressed in a subpopulation of ES cells. We raised a polyclonal antibody against Tcstv1 and confirmed its specificity by the staining pattern, which localized at the membrane in a 2-cell-stage specific pattern of Tcstv1, as reported previously^30^ (Supplementary Fig. 3a). When we stained wild-type ES cells, we found that Tcstv1 expression was detected in ~0.1% of ES cells, which all overlapped with the expression of Rex1-Egfp and Oct3/4-Ecfp in OCRG9 ES cells^31^ (Supplementary Fig. 3b). When *Nr0b1^KO/Y^* ES cells were stained for Tcstv1, the frequency of the Tcstv1-positive cells dramatically increased in the stem cell colonies than in *Nr0b1^fl-/Y^* ES cells (Fig. 4c), suggesting that the increased expression levels of the 2-cell specific transcripts reflected the increase in the subpopulation rather than the increase in their expression levels ubiquitously in the stem cell population.

To investigate the effect of *Nr0b1* on the kinetics of the subpopulation expressing the 2-cell specific transcripts, we applied a *Zscan4c*-promoter-*mCherry* reporter. *Zalzman et al* reported that the 2.5 kb genomic DNA fragment upstream of *Zscan4c* (*Zinc finger and SCAN domain containing 4c*) directed the expression of a fluorescent reporter in ~5% of ES cells and the conversion between the *Zscan4c*-positive subpopulation and the *Zscan4c*-negative subpopulation occurred in a reversible manner^32^. Moreover, depletion of *Zscan4c* by shRNA-mediated knock-down resulted in a crisis of ES cell population within 1 month by shortening of the telomere length, indicating the functional importance of the transient activation of *Zscan4c* in a subpopulation of ES cells^32^. We transfected the *Zscan4c-mCherry* transgene into *Nr0b1^fl-/Y^* and *Nr0b1^KO/Y^* ES cells and three independent cell lines expressing mCherry were established for each genotypes. Observation of these ES cells by fluorescent microscopy revealed a significant increase in mCherry-positive cells in *Nr0b1^KO/Y^*::*Zscan4c-mCherry* ES cells (Fig. 4d). Next we applied FACS analysis to quantify the proportion of the *Zscan4c*-positive subpopulation. In *Nr0b1^fl-/Y^::Zscan4c-mCherry* ES cells, 4.9% of ES cells were mCherry-positive (Fig. 4e), which was comparable to the proportion of *Zscan4c*-positive cells reported previously. In contrast, *Nr0b1^KO/Y^*::*Zscan4c-mCherry* ES cells contained 21.6% of mCherry-positive cells, indicating the dramatic increase in Zscan4c-positive subpopulation, in the same manner found in the immuno-staining for Tcstv1 as shown earlier (Fig. 4c).

Then we performed live-imaging of these ES cells carrying *Zscan4c-mCherry*. As reported previously, *Zscan4c*-positive cells appeared from *Zscan4c*-negative cells (Supplementary Movie 1). The emergence of the Zscan4c-positive cells was much more frequent in *Nr0b1^KO/Y^*::*Zscan4c-mCherry* ES cells than in *Nr0b1^fl-/Y^*::*Zscan4c-mCherry* ES cells, indicating that the increase of *Zscan4c*-positive cells in *Nr0b1^KO/Y^* ES cells was partly due to the altered kinetics in which the conversion from *Zscan4c*-negative to -positive was enhanced. These data suggested that *Nr0b1* negatively regulates the kinetics to generate *Zscan4c*-positive subpopulation.

### *Zscan4c* modulates cell-cycle and cell death

**Figure 5:**
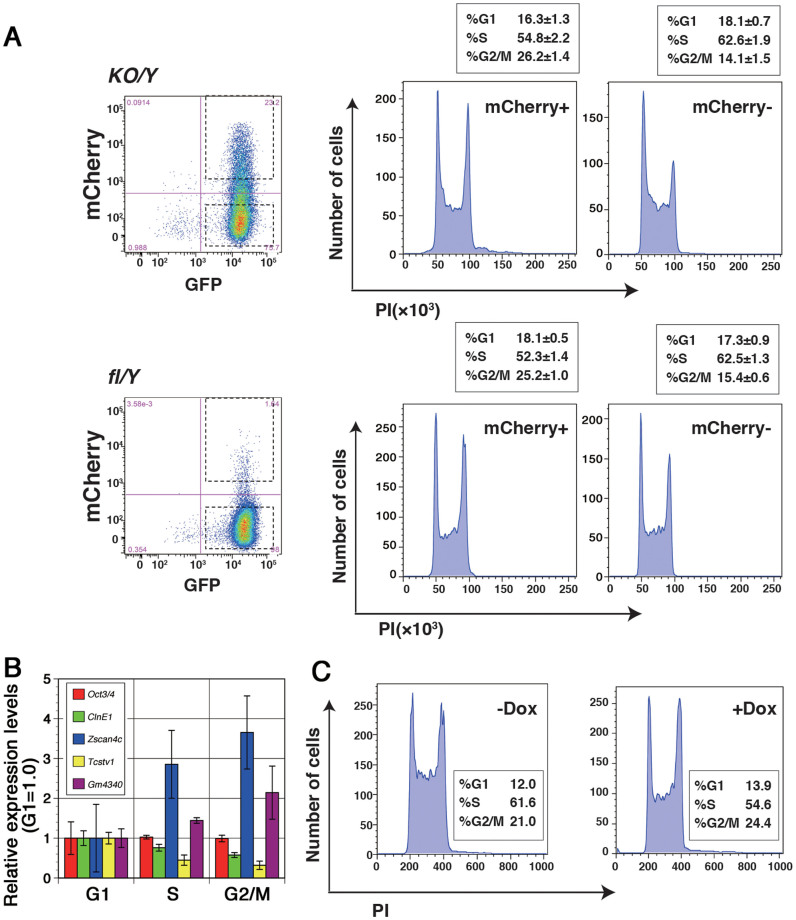
Enrichment of G2 phase by *Zscan4c* expression. (a) Cell-cycle profiles of Zscan4c-mCherry positive and negative cells of *Nr0b1^fl/Y^* (fl/Y) and *Nr0b1^KO/Y^* (KO/Y) ES cells. Each fraction was sorted and analyzed by PI staining. Proportions of the cells at each cell cycle were shown in the box with error values indicating standard deviation (n = 3). (b) Cell-cycle-dependent expression of Zscan4c in ES cells. ES cells at different cell-cycle phases were separaed by FACS based on the Fucci system and the expressions of the indicated genes were quantified by Quantitative RT-PCR. The level of expression of each transcript at G1 phase was set at 1.0. Error bars indicate standard deviation (n = 3). (c) Cell-cycle profiles of ES cells with or without the expression of the Zscan4c transgene. ES cells carrying the doxycline (Dox) inducible Zscan4c transgene were cultured with or without Dox for 2 days and analyzed by PI staining. Proportions of the cells at each cell cycle were shown in the box.

It has been reported that *Zscan4c* is transiently up-regulated in a subpopulation of ES cells to elongate the telomere length via homologous recombination and that the constitutive expression of *Zscan4c* is not tolerated in ES cells^32^,^33^. To investigate the link between the increase in the *Zscan4c*-positive cells and the slow proliferation accompanied by increased G2 phase and cell death in *Nr0b1^KO/Y^* ES cells, we examined the cell-cycle profiles in *Zscan4c*-positive and -negative subpopulations. mCherry-positive and -negative cells were sorted from *Nr0b1^KO/Y^*::*Zscan4c-mCherry* ES cells and their cell-cycle profiles were analyzed by PI staining (Fig. 5a). As a result, we found that the cells at G2 phase were enriched in Zscan4c-positive cells. The similar phenomenon was observed in *Nr0b1^fl-/Y^*::*Zscan4c-mCherry* ES cells, indicating that the Zscan4c-positive cells have increased G2 phase irrespective of the state of *Nr0b1*.

This phenomenon seemed consistent with the function of Zscan4c that mediates homologous recombination to elongate the telomere. To test the direct relation between the expression of *Zscan4c* and the enrichment of G2 phase, we established an ES cell line carrying a tetracycline-inducible *Zscan4c* transgene. These ES cells continue self-renewal in the absence of the inducer. However, once the expression of *Zscan4c* transgene was induced by doxycycline (Dox), these ES cells gradually ceased proliferation as reported previously^34^. We examined the cell-cycle profiles of these ES cells cultured with or without Dox for 2 days and revealed that the ES cells cultured with Dox accumulated at the G2 phase (Fig. 5b). Therefore, the up-regulation of *Zscan4c* could be a cause to limit the transition from G2 to M phase.

To test the link between the activation of *Zscan4c* and the incidence of cell death in a physiological context, we analyzed the live-imaging data of *Zscan4c-mCherry* ES cells. We quantified the total fluorescent intensities of mCherry during one cell cycle, i.e. from the cell division until either the next cell division or cell death. As a result, we found that the cells undergoing cell death expressed higher levels of mCherry than the cells undergoing cell division in both *Nr0b1^fl/Y^* and *Nr0b1^KO/Y^* ES cells (Fig. 4f). These data strongly suggested that up-regulation of *Zscan4c* is a main cause to inhibit proliferation of *Nr0b1^KO/Y^* ES cells via increased incidence of cell death.

### Nr0b1 directly regulates *Zscan4c* expression

**Figure 6:**
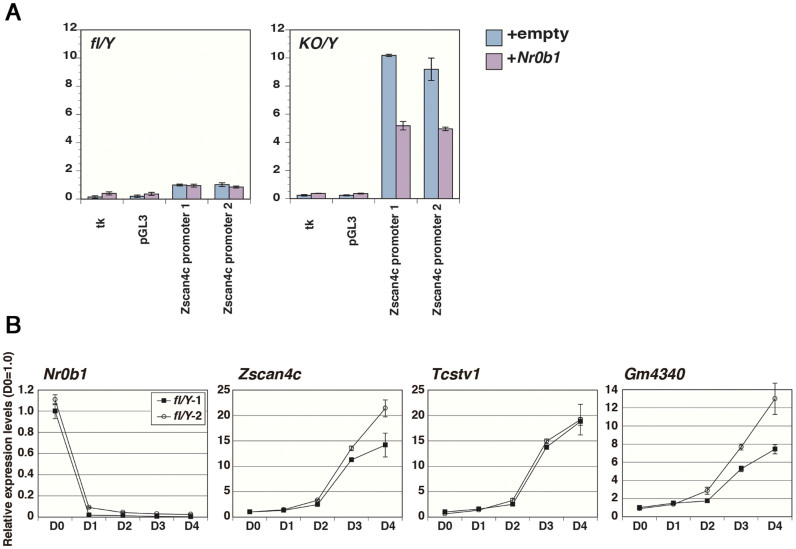
Transcriptional repression of *Zscan4c* by Nr0b1. (a) The activity of the Zscan4c promoter in *Nr0b1^fl/Y^* (fl/Y) and *Nr0b1^KO/Y^* (KO/Y) ES cells. The relative luciferase activity of *pZscan4c-Fluc* was measured by dual-luciferase assay with or without the expression of exogenous Nr0b1. Error bars indicate standard deviation (n = 3). tk (tk-luc) acts as a negative control. (b) Quantitative RT-PCR analysis of *Nr0b1^fl/Y^* (fl/Y) ES cells cultured with Tx for the expressions of 2-cell specific transcript genes. The level of expression of each transcript in *Nr0b1^fl/Y^* ES cells was set at 1.0. Error bars indicate standard deviation (n = 3). The results of two independent experiments were shown.

How does the loss of *Nr0b1* connect to the increase in the subpopulation expressing 2-cell specific transcripts? To determine whether the *Zscan4c*-promoter is directly regulated by Nr0b1, we performed luciferase reporter assay. The *Zscan4c*-promoter was placed upstream of *firefly luciferase* (*Fluc*) and co-transfected with *renilla luciferase* (*Rluc*) under the control of *human cytomegallovious immediate-early* promoter into *Nr0b1^fl-/Y^* and *Nr0b1^KO/Y^* ES cells. Dual luciferase assay of these transfectants demonstrated that the relative Fluc activity driven by the *Zscan4c*-promoter was dramatically increased in *Nr0b1^KO/Y^* ES cells (Fig. 6a), consistent with the results obtained by the *Zscan4c-mCherry* reporter. When these reporters were co-transfected with *Nr0b1* expression vector, the activity of the *Zscan4c*-promoter was significantly repressed in *Nr0b1^KO/Y^* ES cells (Fig. 6a). We also confirmed that these 2-cell specific transcripts including *Zscan4c* were rapidly up-regulated after repression of Nr0b1 by QPCR analysis (Fig. 6b). These data clearly indicated that the *Zscan4c* promoter activity is directly regulated by Nr0b1 in ES cells.

### *Zscan4c* expression is regulated by Nr0b1 in a reversible manner

**Figure 7:**
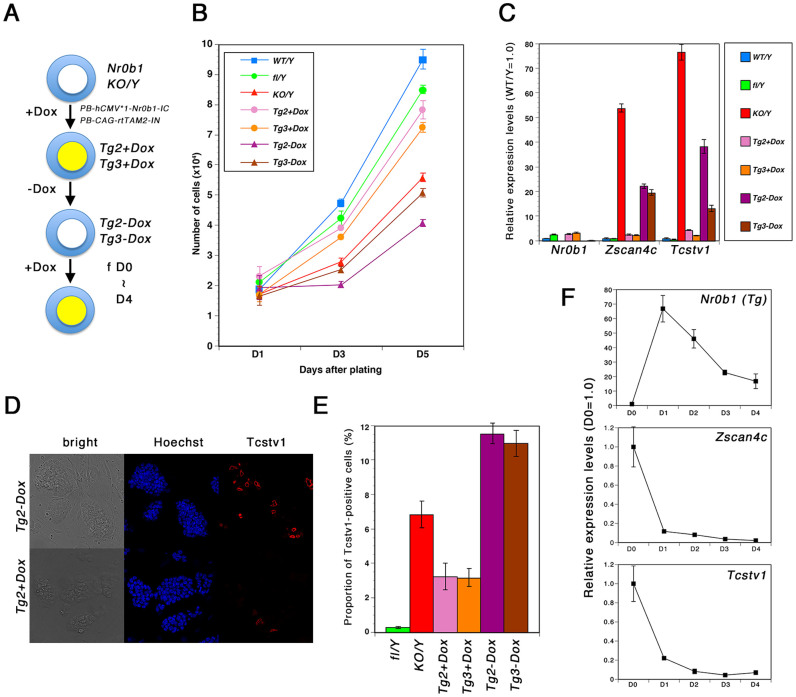
Phenotypic reversion of *Nr0b1*-null ES cells by inducible expression of Nr0b1 transgene. (a) The scheme of the rescue experiments of *Nr0b1*-null ES cells with tetracycline-inducible *Nr0b1* transgene. (b) Proliferation ratio of wild-type (WT), *Nr0b1^fl/Y^* (fl/Y), *Nr0b1^KO/Y^* (KO/Y) and *Nr0b1^KO/Y^* with inducible *Nr0b1*-transgene (clones Tg2 and Tg3) ES cells. 1 × 10^4^ cells were seeded on feeder cells and the numbers of cells were counted after 1, 3 and 5 days. Error bars indicate standard deviation (n = 3). (c) Quantitative RT-PCR analysis of wild-type (WT), *Nr0b1^fl/Y^* (fl/Y), *Nr0b1^KO/Y^* (KO/Y) and Tg ES cells for the expressions of 2-cell specific transcripts. The level of expression of each transcript in *Nr0b1^fl/Y^* ES cells was set at 1.0. Error bars indicate standard deviation (n = 3). (d) Immunostaining of *Tg2 + Dox* and *Tg2 − Dox* ES cells for Tcstv1. Photomicrographs were captured with confocal microscopy. (e) Proportion of Tcstv1-positive cells in Tg ES cells cultured with or without Dox. 10 images captured with confocal microscopy were quantified for each genotype. The average proportions are indicated with standard error. (f) Quantitative RT-PCR analysis of *Tg2-Dox* ES cells cultured with Dox for the expressions of *Nr0b1* transgene and 2-cell specific transcript genes. The level of expression of each transcript in *Tg2-Dox* ES cells was set at 1.0. Error bars indicate standard deviation (n = 3).

To confirm the direct regulation of *Zscan4c* by Nr0b1, we established *Nr0b1^KO/Y^* ES cell lines carrying tetracycline-inducible *Nr0b1* transgene by introduction of pPB-hCMV*-1-Nr0b1-IRES-mCherry and *pPB-CAG-rtTAM2-IN* followed by the selection with G418 in the presence of Dox (Fig. 7a). We isolated the colonies with rapid proliferation and analyzed their characters. These Nr0b1^KO/Y^::hCMV*1-Nr0b1 ES cells proliferated faster with lower levels of *Zscan4c* and *Tcstv1* expression than in *Nr0b1^KO/Y^* ES cells (Fig. 7b and 7c). Then we tested the expression of Nr0b1 protein by western blot. As shown in Supplementary Fig. 4, Nr0b1 expression in Nr0b1^KO/Y^::hCMV*1-Nr0b1 ES cells was significantly lower than that in *Nr0b1^fl/Y^* ES cells, which might be a reason why these ES cells still showed differences from *Nr0b1^fl/Y^* ES cells in proliferation speed as well as the expression of 2-cell marker genes. We next examined the impact of *Nr0b1* expression on the proportion of 2-cell subpopulation. We evaluated the proportion of Tcstv1-positive cells in Nr0b1^KO/Y^::hCMV*1-Nr0b1 ES cells cultured with or without Dox by immunostaining and found that the 2-cell subpopulation was decreased when *Nr0b1* transgene was activated (Fig. 7d and 7e). The proportion of Tcstv1-positive cells as well as the expression of 2-cell marker genes were reverted to high levels when these ES cells were cultured without Dox (Fig. 7c and 7e), indicating that the expression of 2-cell markers are regulated in a *Nr0b1*-dependent manner. Moreover, by the activation of *Nr0b1* with Dox, the expression of 2-cell marker genes were repressed within 24 hours after the addition of Dox. These data confirmed that the 2-cell marker genes including *Zscan4c* are direct targets of Nr0b1.

### Knockout of *Nr5a2* causes similar phenotype to knockout of *Nr0b1*

Nr0b1 lacks the DNA binding domain and acts as a partner of other nucelar receptor family members. Nr5a2 is a strong candidate partner of Nr0b1 from previous publications^9^,^10^. To test the role of *Nr5a2* in ES cells, we made inducible knocout ES cells of *Nr5a2* using the Cre-*loxP* system as in the case of Nr0b1 (figure S5a). As a result, we found that induced knockout of *Nr5a2* also caused the induction of 2-cell markers (figure S5c) with reduced proliferation (figure S5b). These data suggested that Nr5a2 might be a partner of Nr0b1 and the heterodimer acts as a transcriptional repressor of 2-cell marker genes including *Zscan4c*.

## Discussion

Mouse ES cells continue self-renewal *in vitro* in an unlimited manner^34^. To continue proliferation, the telomere length should be actively maintained because it shortens along the cycle of chromosome replication and its shortening results in chromosome instability and cell lethality^35^. The elongation of the telomere by the enzymatic activity of telomerase is regarded as a major pathway to keep the telomere length. The catalytic activity of telomerase is encoded by *Telomerase reverse transcriptase* (*Tert*). *Tert* is expressed in many proliferating cells including stem cells. However, it was reported that the ES cells lacking *Tert* generated by gene-targeting continue proliferation with gradual shortening of the telomere length and finally reaches the crisis after the culture over 1 year, indicating that there are other systems to keep the telomere length in ES cells^36^,^37^,^38^. In 2010, *Zalzman et al* reported a novel mechanism to maintain the telomere length in mouse ES cells^32^. They focused on the functional analysis of *Zscan4c* in ES cells because they had found that *Zscan4c* is transcriptionally activated in the late 2-cell stage in mouse pre-implantation embryos and shows a salt-and-pepper expression pattern in ES cells. When they knocked down *Zscan4c* in ES cells, these ES cells stopped proliferation after 8–9 passages within 1 month by shortening the telomere length. They found that Zscan4c co-operates with Rad50 and Mre11 to maintain the telomere length by homologous recombination as found in some cancer cells. The regulation of *Zscan4c* expression is quite unique since only 5% of ES cells express it at a certain time point but all of the expression is reversible and transient, and all ES cells in the culture experience the expression of *Zscan4c* within 1 month to maintain the telomere length. It has remained unclear how this interesting regulation is achieved, but here we found that *Nr0b1* is involved in the regulation of the *Zscan4c* expression.

In ES cells, Zfp206/Zscan10 was previously identified as the transcriptional activator of *Zscan4c*^25^. *Zscan4c* and other 2-cell specific transcripts were down-regulated in *Zfp206* knock-down ES cells and *Zfp206* was transiently induced at early 2-cell stage preceding the induction of 2-cell specific transcripts in pre-implantation development. However, our microarray analysis indicated that the expression of *Zfp206* was not affected by elimination of *Nr0b1* (Fig. 4b), suggesting that the regulation of 2-cell specific transcripts by Nr0b1 is independent of *Zfp206*. It was also reported that *Zscan4c* is repressed by the inhibition of the phosphatidylinositol-3-OH kinase (PI3K)-Akt pathway in ES cells^39^. However, *Nr0b1* was also down-regulated in this condition, which is inconsistent with our present observation. Recently, Macfarlan et al demonstrated that ES cells possess a subpopulation expressing endogenous retrovirus as well as 2-cell specific transcripts and their expressions were antagonized by repressive chromatin-modifying enzymes such as Kdm1a, Kap1 and G9a^40^. However, none of these genes were up-regulated in *Nr0b1^KO/Y^* ES cells (Fig. 4b). More recently, Dan et al reported that Rif1 directly regulates *Zscan4c* expression by mediating epigenetic repression^41^. Interestingly, the phenotype of Rif1 knockdown ES cells is quite similar to that of *Nr0b1* knockout ES cells reported in this manuscript such as slow proliferation with increased proportion G2/M phase and induction of 2-cell marker genes. However, *Nr0b1* is not included in the list of the genes down-regulated in *Rif1* knockdown ES cells and *Rif1* expression is not repressed in our microarray data of *Nr0b1* knockout ES cells (Fig. 4b). Therefore, the negative regulation of *Zscan4c* by Nr0b1, which was in fact a direct repression, is unique from the previous observations.

We demonstrated that *Zscan4c* is a functional target of *Nr0b1*. The *Nr0b1*-null ES cells proliferate slowly with accumulation at G2/M phase and high incidence of apoptosis. Over-expression of *Zscan4c* also induced the similar phenotype efficiently, and the increased proportion of *Zscan4c*-positive cells found in *Nr0b1^KO/Y^* ES cells accords to explain the phenotype. Moreover, our live-imaging analysis of *Zscan4c-mCherry* ES cells revealed that the cells undergoing cell death show higher levels of *Zscan4c* activity than the cells undergoing cell division (Fig. 4f). Therefore, the aberrant expression of *Zscan4c* could be a primary cause of the phenotype observed in *Nr0b1^KO/Y^* ES cells. We also found using an inducible knockout line of *Nr5a2* that Nr5a2 is a strong candidate partner of Nr0b1 to repress *Zscan4c* expression. The functional link of Nr0b1:Nr5a2 and *Zscan4c* confers the connection between the pluripotency-associated transcription factor network and the cell-biological character of ES cells, i.e. the unlimited ability to self-renew. However, since *Zscan4c* was not expressed in all *Nr0b1^KO/Y^* ES cells, the repression of *Zscan4c* by *Nr0b1* is only a part of the regulatory mechanism. Further analysis will be required for revealing the unique mechanism that induces *Zscan4c* expression in a small subpopulation transiently.

Several reports pointed the role of *Nr0b1* in maintaining pluripotency. Two preceding papers demonstrated the essential role of *Nr0b1* in self-renewal of ES cells by loss-of-function analyses^12^,^13^. However, here we succeeded in establishing *Nr0b1*-null ES cells. siRNA-mediated knock-down strategy sometimes show severer phenotypes than those observed by gene-targeting, which might be due to the faster kinetics of ablation of the gene function without the time for compensation or the off-target effect. Here we applied the inducible knockout using the Cre-*loxP* system, providing a rapid kinetics of the functional ablation of *Nr0b1*. However, most of the knockout ES cells continued self-renewal, which allowed efficient establishment of *Nr0b1*-null ES cells. The transcriptomic analysis suggested that there was no remarkable change in the gene expression pattern to adopt the loss of *Nr0b1* for continuous self-renewal (data not shown). Therefore, the discrepancy from the previous reports might be due to the off-target effect of knock-down strategies applied in the previous reports otherwise the reason for the different result obtained by the inducible knockout remains unclear. We observed enhancement of spontaneous differentiation in *Nr0b1^KO/Y^* ES cells although the majority of these ES cells continued self-renewal with slow proliferation ratio. It could be true that, these ES cells might have higher incidence to loose proper pluripotency. We observed that both *Nr0b1^fl-/Y^* ES cells and *Nr0b1^KO/Y^* ES cells established in the culture without feeder cells lost the ability to contribute to chimeric embryos after injection into blastocysts with over-expression of *Klf4* and *Tbx3* (data not shown). The role of the feeder cells to settle the over-expression of *Klf4* and *Tbx3* is obscure, but it may suggest the role of *Nr0b1* in stabilizing the pluripotency-associated transcription factor network. Although its function looks not essential to maintain pluripotency as suggested by the comprehensive analyses of the network, here we disclosed the definitive function of *Nr0b1* on the regulation of 2-cell specific transcripts to limit their expression in a small subpopulation. Further analysis will reveal the role in the pluripotency-associated transcription factor network that controls pluripotency in biological sense.

## Methods

### Cell culture

EB5 ES cells (derived from male E14tg2a ES cells) were cultured with or without mouse embryonic fibroblast feeder cells treated with Mitomycin C in GMEM supplemented with 10% FCS, 1× sodium pyruvate, 1× non-essential amino acids, 10^−4^ M of 2-mercaptoethanol and 1000 U/ml of LIF.

### Plasmid construction

For generation of *Nr0b1* KO vector, genomic DNA fragments for 5′ and 3′ homology arms (Chr:X, 86191214-86195211 and 86196711-86199810 in GRCm38) as well as the floxed region containing exon 2 (Chr:X, 86195212-86196710 in GRCm38) were amplified from EB5 genomic DNA using the primer pairs Nr0b1-5′, Nr0b1-3′ and Nr0b1-flox, respectively, shown in Suplementary Table 1. The 5′ arm and the floxed region were digested with ClaI and NotI, respectively, and inserted into ClaI-NotI of *pBR-MC1DTApA* using ligation and In-fusion cloning (Clonetech), resulting in *pDTA-Nr0b1-5′+flox*. Then the 3′ arm was digested by BglII and NotI and inserted into BglII and NotI of *pDTA-Nr0b1-5′+flox*, resulting in *pDTA-Nr0b1-5′+flox+3′*. Finally, the *Frt-PGKpacΔtkpA-Frt* cassette was introduced into the BglII site of *pDTA-Nr0b1-5′+flox+3′*, resulting in *pDTA-Nr0b1 floxKO*.

The PiggyBac vectors *pPBCAG-MerCreMer-IH*, *pPBCAG-Egfp-IZ*, *pPBCAG-mCherry-IP*, *pPBCAG-rtTAM2-IN* and pPB-hCMV*-1-IRES-mCherry-pA^42^,^43^,^44^ were constructed based on *pGG131*^45^. For construction of the *Zscan4c* reporters, the *Zscan4c* promoter (Chr:7, 11003915-11006554 in GRCm38) was amplified from EB5 genomic DNA using KOD-Plus-Neo (TOYOBO) using the primer pair Zscan4c-promoter shown in Supplementary Table 1 and subcloned into *pPBCAG-mCherry-IP* by replacing *CAG* promoter and SacI-XhoI of pGL3-Basic (Promega), resulting in *pPB-Zscan4c-mCherry* and *pZscan4c-Fluc*, respectively. For inducible expression of *Zscan4c*, the entire coding region was amplified with the primer pair Zscan4c-CDS from EB5 cDNA and subcloned into pPB-hCMV*-1-IRES-mCherry-pA, resulting in pPB-hCMV*-1-Zscan4c-IRES-mCherry. The expression vector of *Nr0b1* was generated by insertion of the entire coding region of *Nr0b1* amplified from EB5 cDNA with the primer pair Nr0b1-CDS into *pPyCAG-IP*, resulting in *pPyCAG-Nr0b1-IP*. For inducible expression of Nr0b1, the XhoI-NotI fragment of Nr0b1 CDS was subcloned into pPB-hCMV*-1-IRES-mCherry-pA, resulting in pPB-hCMV*-1-Nr0b1-IRES-mCherry.

For generation of *Nr5a2* KO vector, genomic DNA fragments for 5′ and 3′ homology arms (Chr:1, 136951766-136950374 and 136944805-136943464 in GRCm38) as well as the floxed region containing exons 3 and 4 (Chr:1, 136950373-136944806 in GRCm38) were amplified from EB5 genomic DNA using the primer pairs Nr5a2-5′, Nr5a2-3′ and Nr5a2-flox, respectively, shown in Suplementary Table 1. These PCR fragments were assembled in EcoRV site of pBR-blue II, resulting in pBR-Nr5a2 5′+flox+3′. Finally, the Frt-SA-Ires-neo-pA:PGKpacΔtkpA-Frt cassette was introduced into the BamHI site flanking the 5′ end of the 3′ loxP sequence of pBR-Nr5a2 5′+flox+3′, resulting in pBR-Nr5a2 floxKO.

### Generation of inducible *Nr0b1*-null and Nr5a2-null ES cells

10^7^ EB5 ES cells were electroporated with 100 μg of linealized *Nr0b1* KO vector DNA at 800 V and 3 μF in a 0.4-cm cuvette using a Gene Pulser (Bio-Rad), followed by culture with 1.5 μg/ml of puromycin for 8 days. The resulting stem cell colonies were picked up, expanded and genotyped by PCR using the primer pairs KO-PCR1 and KO-PCR2. The correctly targeted clones (*Nr0b1^flFRT/Y^*) were seeded in a well of 48-well plate at 10^4^ cells per well, and transfected with 1 μg of circular *pCAG-FLPe-IP* plasmid using Lipofectoamine 2000 (Invitrogen) followed by the culture for 3 days. Then these transfected cells were replated and cultured with 1 μM of Gancyclovir for 8 days. The resulting stem cell colonies were picked up, expanded and genotyped by PCR using the primer pair Nr0b1-flox. The clones in which the *PGKpac*Δ*tkpA* cassette flanked by *Frt* were correctly removed (*Nr0b1^fl-/Y^*) were seeded in a well of 48-well plate at 10^4^ cells per well, and transfected with 0.25 μg of circular *pPB-CAG-MerCreMer-IH*, 0.25 μg of circular *pPB-CAG-Egfp-IZ*, and 0.5 μg of circular *pCAG-PiggyBac transposase* (*PBase*) plasmid using Lipofectoamine 2000 followed by the culture for 3 days. Then these transfected cells were replated and cultured with 200 μg/ml of Hygromycin B and 20 μg/ml of Zeocin for 8 days. The resulting stem cell colonies were picked up, expanded and assessed for the expression of Egfp by fluorescent microscopic analysis as well as the function of MerCreMer by PCR genotyping of the Tx-treated cells using the primer pair P1. Finally, two independent pairs of *Nr0b1^flFRT/Y^* and its descendant *Nr0b1^KO/Y^* ES cells were analyzed in this study. For monitoring the expression of *Zscan4c*, *Nr0b1^flFRT/Y^* and *Nr0b1^KO/Y^* ES cells were seeded in a well of 48-well plate at 10^4^ cells per well, and transfected with 0.5 μg each of pPB-Zscan4c-mCherry and pCAG-PBase, and 0.05 μg of *pPB-CAG-Egfp-IZ* plasmid using Lipofectoamine 2000 followed by the culture for 3 days. Then these transfected cells were replated and cultured with 20 μg/ml of Zeocin for 8 days. The resulting stem cell colonies were picked up, expanded and assessed for the expression of mCherry by fluorescent microscopic analysis. To establish ES cells carrying the inducible Zscan4c, ES cells were seeded in a well of 48-well plate at 10^4^ cells per well, and transfected with 0.5 μg each of pPB-hCMV*-1-Zscan4c-IRES-mCherry and *pCAG-PBase*, and 0.05 μg of *pPB-CAG-rtTAM2-IN* plasmid using Lipofectoamine 2000 followed by the culture for 3 days. Then these transfected cells were replated and cultured with 160 μg/ml of G418 for 8 days. The resulting stem cell colonies were picked up, expanded and assessed for the expression of mCherry in the presence of Doxycycline (1 μg/ml) by fluorescent microscopic analysis. Establishment of *Nr0b1*-null ES cells carrying the inducible *Nr0b1* was performed in the same way.

The inducible Nr5a2 KO ES cells were generated in the same way as the inducible Nr0b1 KO ES cells except for the serial KO of the second allele.

### Production of chimeric embryos

Dissociated ES cells were injected into a C57BL6 blastocyst by microinjection, which was then transferred into the uterus of a pseudopregnant female ICR mouse. Embryos were collected at 13.5 dpc to evaluate chimera contribution ability of ES cells by analyzing with fluorescence microscopy. All animal experiments confirmed to our Guidelines for the Care and Use of Laboratory animals and were approved by the Institutional Committee for Laboratory Animal Experimentation (RIKEN Kobe Institute).

### Immunostaining and Western blot

Polyclonal antibody against Tcstv1 was obtained by immunization of rabbit by purified fusion protein of GST and full-length Tcstv1 followed by the purification of antibody by affinity column. Cells were fixed with 4% of paraformaldehyde for 30 minutes at 4°C, washed with PBS containing 2% FCS, and incubated with anti-Tcstv1 antibody (1:1000) for overnight at 4°C. After washing with PBS, the cells were incubated with donkey anti-rabbit IgG Alexa-Fluor-594-conjugated antibody (Molecular Probes) for 30 minutes at room temperature. Fluorescent images were captured using an IX51 microscope with DP70 digital camera (Olympus) or a Leica SP2 confocal microscope (Leica). Western blot was performed with anti-Nr0b1 (Active Motif, #39983) and anti-Cdk2 (Santa Cruz, sc-163) for the total cell lysate of wild-type, *Nr0b1^flFRT/Y^* and *Nr0b1^KO/Y^* ES cells.

### FACS analysis

Cell-cycle analysis were done by staining cells with propidium iodide (SIGMA-ALDRICH) in 2 different experimental steps. As the first step, *Nr0b1^flFRT/Y^* and *Nr0b1^KO/Y^* ES cells were cultured in GMEM/10%FCS/LIF medium and cell-cycle status were measured. The second step is that cherry positive/negative cells were sorted from Zscan4-mCherry expressing *Nr0b1^flFRT/Y^* and *Nr0b1^KO/Y^* cells and analyzed.

For apoptotic assay, Annexin V-APC (BD Biosciences) staining was performed according to the manufacturer's protocol using *Nr0b1^flFRT/Y^* and *Nr0b1^KO/Y^* ES cell. Apoptotic cells were assessed by FACS (BD Biosciences) and analyzed by Flowjo software (Digital biology).

### Real-time PCR analysis

First strand DNA was synthesized from 500 ng of total RNA prepared by QuickGene RNA cultured cell HC kit (KURABO) in 20 μl of the reaction mixture containing oligo-dT primers using a ReverTra Ace first strand synthesis kit (TOYOBO). Real-time PCR was performed with THUNDERBIRD SYBR qPCR Mix (TOYOBO) using CFX384 Real-Time System (Bio-Rad). Sequences of primer pairs are listed in Supplementary Table 1.

### Microarray analysis

DNA microarray analyses were performed using a SurePrint G3 Mouse GE Microarray 8 × 60 K (Agilent Technologies). Microarray results were analyzed using NIA Array Analysis Software. Complete array data will be available on the GEO (NCBI) website.

### Live cell imaging

For live cell imaging, 3 clones of each *Nr0b1^KO/Y^*::*Zscan4c-mCherry* and *Nr0b1^fl-/Y^*::*Zscan4c-mCherry* ES cells were seeded 1000cells per well on a ibidi 8well chamber (Nippon Genetics) coated with poly-L-lysin (100 μg/ml; Sigma-Aldrich) and then with Ecadherin-Fc (5 μg/ml; R&D Systems). Cells were monitored in a humid atmosphere with 5% CO_2_ at 37°C under an inverted microscope (IX81, Olympus) equipped with a confocal spinning disk (CSU-X1, Yokogawa), a CCD camera (iXon, Andor) and MetaMorph imaging software (Molecular Devices). Time lapse images were taken every 1 hour with a 10× objective lens.

### Luciferase assay

For assessing the *Zscan4c* promoter activity in *Nr0b1^flFRT/Y^* and *Nr0b1^KO/Y^* ES cells, 6 × 10^4^ cells were seeded in each well of a 24-well plate and transfected with 0.4 μg of circular *pZscan4c-Fluc*, 1.6 μg of circular *pPyCAG-IP* or *pPyCAG-Nr0b1-IP*, and 0.04 μg of circular *pCMV-Rluc* plasmid (Promega) using Lipofectoamine 2000 followed by the culture for 24 hours. Luciferase assays were performed using a Dual-luciferase assay kit (Promega).

## Supplementary Material

Supplementary Information Supplementary information

Supplementary Information Supplementary Movie 1
