# Supplementary material for: Nr0b1 is a negative regulator of Zscan4c in mouse embryonic stem cells
Source: Sci Rep. 2015 Mar 16;5:9146. doi: 10.1038/srep09146 (PMC5390923; doi:10.1038/srep09146)
Supplement: Supplementary Information [file srep09146-s1.pdf]

Supplementary information

Nr0b1 is a negative regulator of *Zscan4c* in mouse embryonic stem cells

Setsuko Fujii, Satomi Nishikawa-Torikai, Yoko Futatsugi, Yayoi Toyooka, Mariko Yamane, Satoshi Ohtsuka and Hitoshi Niwa

Legends for supplemental data

Supplemental Figure 1. (related to Fig. 1) Western blot analysis of Nr0b1 expression in wild-type (+/Y), *Nr0b1*$^{fl/Y}$ (fl/Y) and *Nr0b1*$^{KO/Y}$ (KO/Y) ES cells. Data from two independent *Nr0b1*$^{fl/Y}$ (fl/Y) ES cell lines were shown and left set was shown in Fig 1d.

Supplemental Figure 2. (related to Fig. 4) Immunostaining of *Nr0b1*$^{KO/Y}$ ES cells by antibodies against Gata4 (C-20, Santa Cruz), Sox7 (R&D) and Sox17 (R&D); left: bright field, center: Hoechst staining, right: antibody staining detected by Alexa 594.

Supplemental Figure 3. (related to Fig. 4) Immunostaining by anti-Tcstv1 antibody. (a) Immunostaining of mouse pre-implantation embryos; green: Tcstv1, red: Oct3/4. (b) Immunostaining of OCRG9 ES cells; red: Tcstv1, green: Rex1Egfp, cyan: Oct3/4Ecfp, blue: Hoechst staining.

Supplemental Figure 4. (related to Fig. 7) Western blot analysis of Nr0b1 expression in wild-type (+/Y), *Nr0b1*$^{fl/Y}$ (fl/Y) *Nr0b1*$^{KO/Y}$ (KO/Y) ES cells, and Tg ES cells cultured with or without Dox. CDK2 acts as a control.

Supplemental Figure 5. Generation and analysis of inducible *Nr5a2*-null ES cells. (a) Schematic representation of the strategy to generate the inducible *Nr5a2*–null ES cells.

(b) Colony morphologies of *Nr5a2*$^{fl/fl}$ ES cells carrying hormone inducible Cre after the culture for 5 days without (upper panel) or with Tx (lower panel). Scale bar; 200 μm.

(c) Quantitative RT-PCR analysis of inducible *Nr5a2*–null ES cells cultured with or without Tx for 5 days. The expressions of pluripotency-associated genes (upper panel) and 2-cell specific transcript genes (lower panel) are indicated. The expression levels of each transcript in inducible *Nr5a2*-null ES cells without Tx was set at 1.0. Error bars indicate standard deviation (n=3).

Supplemental Table 1. List of the primer sequence

Supplemental Table 2. Efficiency of the production of chimeric embryos from *Nr0b1*$^{fl/Y}$ and *Nr0b1*$^{KO/Y}$ ES cells carrying *pPBCAG-Egfp-IP*. Embryos carrying GFP-positive cells were designated as chimeras.

Supplemental Movie 1. Live-imaging of the fluorescent signal of *Nr0b1*$^{fl/Y}$ and *Nr0b1*$^{KO/Y}$ ES cells carrying *pPB-Zscan4c-mCherry*.

Supplemental Fig 1

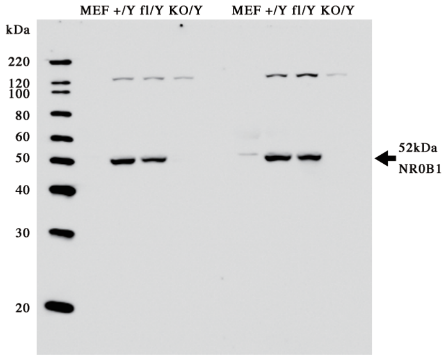

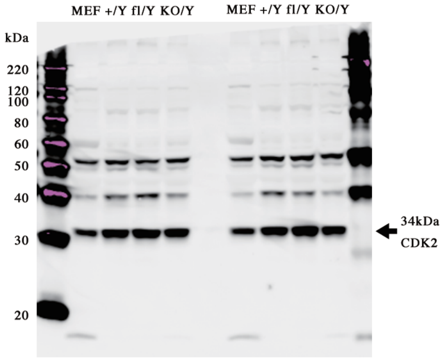

Supplemental Fig 2

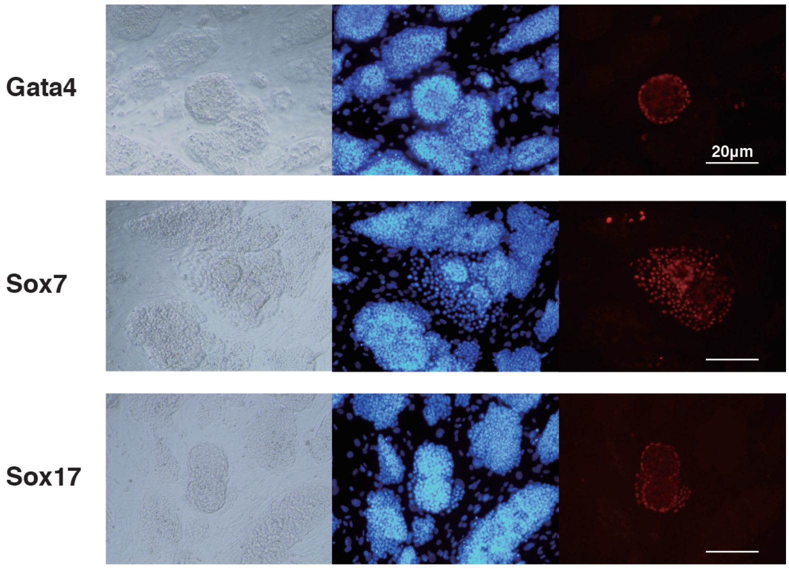

Supplemental Fig 3

A

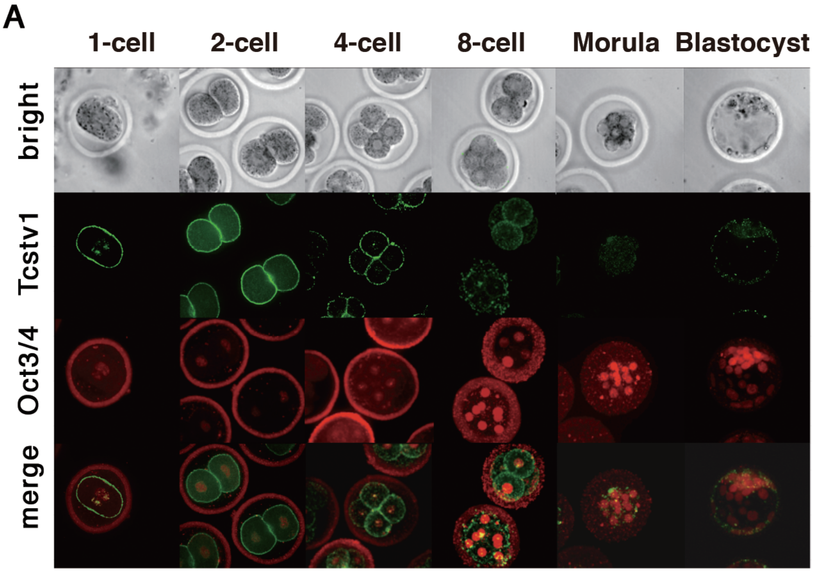

B

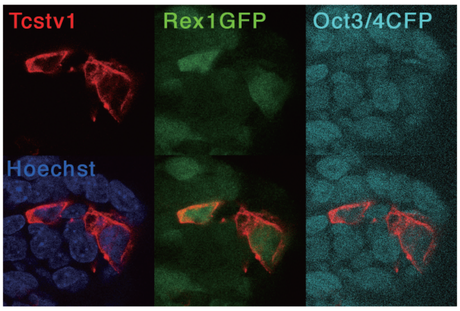

Supplemental Fig 4

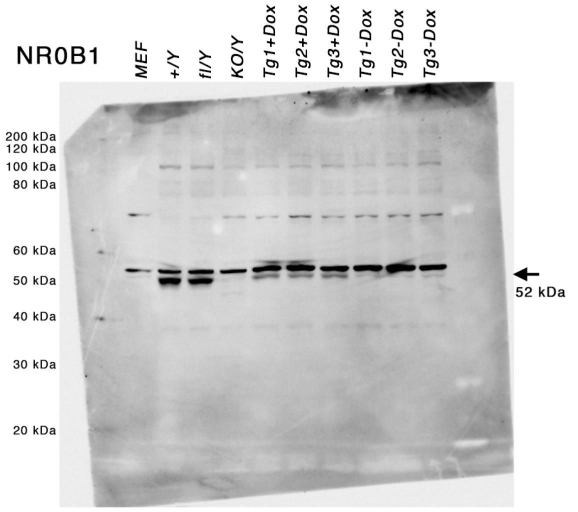

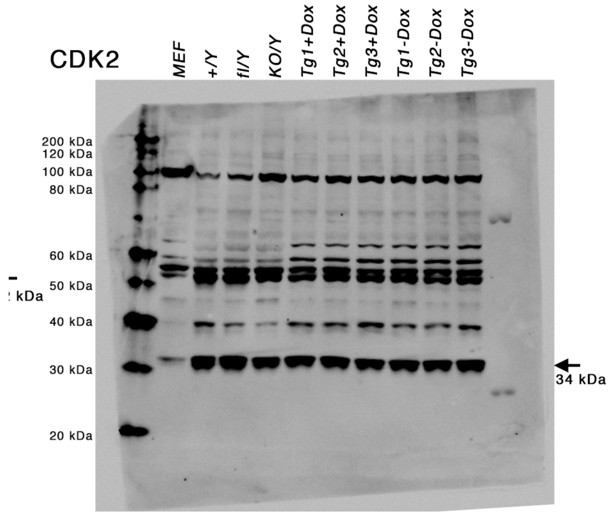

Supplemental Fig 5

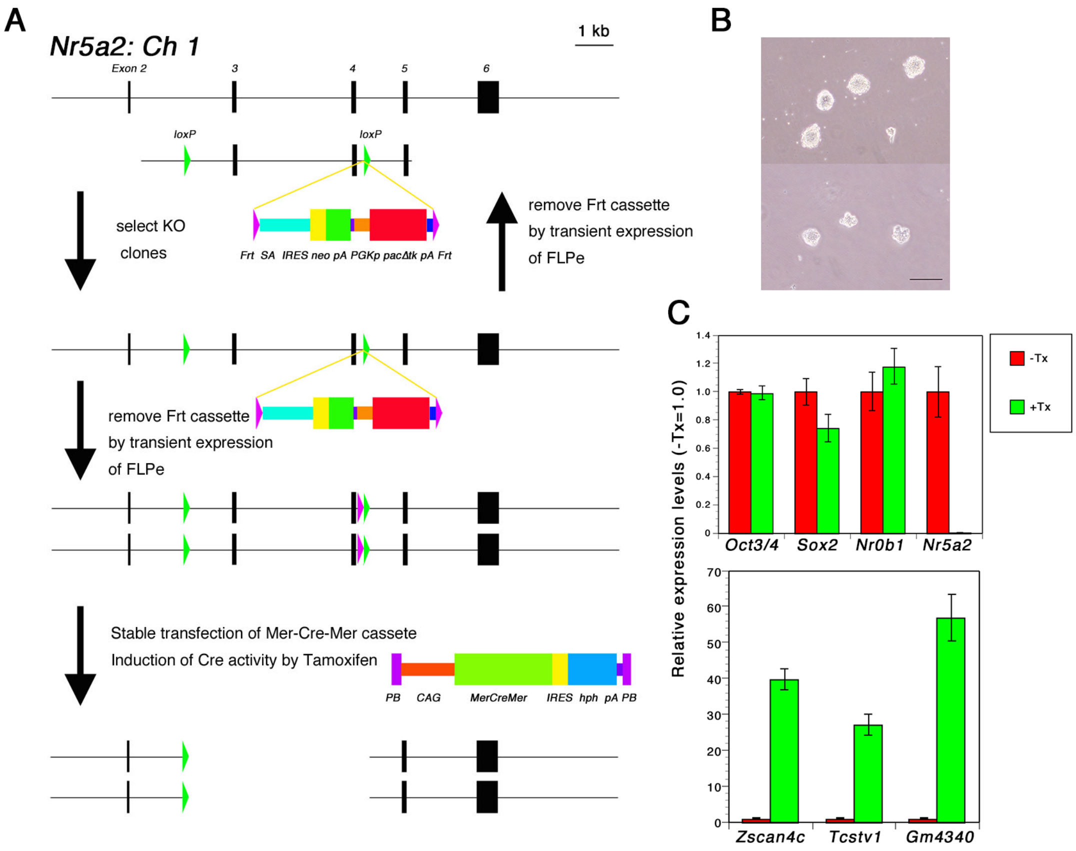

Table S1. List of primer sequence.

For plasmid construction

Target gene	Forward primer	Reverse primer
Nr0b1 (5' arm)	5'-gctcgacggtatcgatactcacact cagatagcttggctagc -3'	5'-taatgtatgctatacgaagttatatggac tgaaataagttctaagttttcagaagca -3'
Nr0b1 (flox)	5'-cgtatagcatacattatacgaagtta ttattttaggccccagaaaaccccttt -3'	5'-gtataatgtatgctatacgaagttatattc acataaagatgtttaaaactctgttgag-3'
Nr0b1 (3' arm)	5'-tgaagatctccttagaagcctggtg aatc -3'	5'-aatgcggccgctgatgaattcatccac attctgcct -3'
Nr0b1 (KO PCR 1)	5'-gcgacggtgacgaaggccagcaa ttagg -3'	5'-cggaataggaacttcagatctcgatcta ta -3'
Nr0b1 (KO PCR 2)	5'-aagtgttgcttctgaaaacttagaac ttatttcagtccatataacttcgt -3'	5'-tgcactcacatgcacagacacacaca cacg -3'
Zscan4c (promoter)	5'-attgtcgacaccttctgttcatgcaag cctgg -3'	5'-aataccggtctcgagtggtgacaatggt gtgaaagattc -3'
Zscan4c (CDS)	5'-attctcgagtcaccacaatggcttca cagcaggca -3'	5'-aatgcggccgcgttcagtcagatctgtg gtaattcctc -3'
Nr0b1 (CDS)	5'-ccctcgagcctcaggccatggcgg gtgaggaccaccgt -3'	5'-ccgcggccgctcacagctttgcacaga acatc -3'

For QPCR

Target gene	Forward primer	Reverse primer
Gapdh	5'-tgccatcactgccacccagaagactg -3'	5'-tgaggtccaccaccctgttgctgtag -3'
Oct3/4	5'- aagccctccctacagcagat -3'	5'- ctgggaaaggtgtccctgta -3'
Nr0b1	5'- tccaggccatcaagagtttc -3'	5'- atctgctgggttctccactg -3'
Sox2	5'- gagtggaaacttttgtccgaga -3'	5'- gaagcgtgtacttatccttcttcat -3'
Nanog	5'-acctgagctataagcaggttaagac -3'	5'- gtgctgagcccttctgaatcagac -3'
Tbx3	5'- tcccggaaacagaattcatc -3'	5'- gagacagcaggagaggatgc -3'

Klf2	5’- accaagagctcgcacctaaa -3’	5’- gtggcactgaaagggtctgt -3’
Klf4	5’- ccaaagaggggaagaaggtc -3’	5’- cctgtgtgtttgcggtagtg -3’
Klf5	5’-caagccgttccagtgcat-3’	5’-gtctgcggtttaaaggatgg-3’
Esrrb	5’-ctcgccaactcagattcgat-3’	5’-agaagtgttgcacggctttg-3’
Zscan4c	5’-ccggagaaagcagtgaggtgga-3’	5’-cgaaaatgctaacagttgat-3’
Tcstv1	5’-tccccaggagatgtggaaaa-3’	5’-cgtcacaggtcacaagatccaga-3’
Gm4340	5’-tggacttccattcacagcttc-3’	5’-catggaatttggccttgtatc-3’
Nr5a2	5’-agatgccagaaaacatgcaa-3’	5’-tatcgccacacacaggacat-3’
ClnE1	5’-ttctgcagcgtcatcctct-3’	5’-tggagcttatagacttcgcaca-3’

Table S2. Efficiency of the production of chimeric embryos.

Cell line	Number of collected embryos	Number of chimeras
Nr0b1$^{Floxed/Y}$ 1	7	0
Nr0b1$^{Floxed/Y}$ 2	12	4
Nr0b1$^{Floxed/Y}$ 3	4	0
Nr0b1$^{Null/Y}$ 1	10	1
Nr0b1$^{Null/Y}$ 2	6	0
Nr0b1$^{Null/Y}$ 3	13	1
Nr0b1$^{Null/Y}$ 4	6	2
